# Integrated acoustic identification of a petit-spot volcanic field in the oldest Pacific plate

**DOI:** 10.1038/s41598-025-15806-y

**Published:** 2025-09-09

**Authors:** Shiki Machida, Junji Kaneko, Kazuhiro Inose, Tatsuo Nozaki, Koichi Iijima, Naoto Hirano

**Affiliations:** 1 Ocean Resources Research Center for Next Generation (ORCeNG), Chiba Institute of Technology, 2-17-1 Tsudanuma, Narashino, 275-0016 Chiba Japan; 2 Japan Agency for Marine-Earth Science and Technology (JAMSTEC), 2-15 Natsushima-cho, Yokosuka, 237-0061 Kanagawa Japan; 3 Viziotex Corporation, 568-14 Marubayashi, Shimotsuga Nogi-machi, Tochigi, 329-0111 Japan; 4 Faculty of Science and Engineering, Waseda University, 3-4-1 Okubo, Shinjuku-ku, Tokyo, 169-8555 Japan; 5 Frontier Research Center for Energy and Resources, School of Engineering, The University of Tokyo, 7-3-1 Hongo, Bunkyo-ku, Tokyo, 113- 8656 Japan; 6 Center for Northeast Asian Studies, Tohoku University, 41 Kawauchi, Sendai Aoba-ku, Miyagi, 980-8576 Japan

**Keywords:** Petit-spot, Subbottom profiler, Multibeam echo sounder, Volcanic field, Pacific ocean, Geology, Volcanology

## Abstract

Petit-spot volcanism plays a critical role in the metasomatism of oceanic plates prior to subduction and in their recycling into the deep mantle. The extent of metasomatism depends on the number and volume of petit-spot volcanic edifices and intrusions, making precise identification of petit-spot volcanic fields essential. However, conventional methods based on seafloor topography and acoustic backscatter intensity alone have limitations in accurately delineating these features. Here we compiled acoustic survey data obtained using subbottom profilers mounted on both research vessels and submersibles in the region southeast of Minamitorishima (Marcus) Island, the oldest part of the Pacific plate. Analysis of the subbottom profiler data revealed three main stratigraphic facies: O-type (opaque), T-type (transparent), and L-type (layered). Among these types, O_L_-type facies, characterized by low slope gradients and high backscatter intensity, indicate petit-spot lava flows. Additionally, M-type facies, marked by multiple discontinuous reflections, occur at boundaries between O_L_-type and other facies, representing transitional zones formed by intrusive activity. High-resolution acoustic stratigraphy and ground-truth observations from submersible dives confirmed that M-type facies reflect petit-spot lava intrusions. Integrating seafloor backscatter with subseafloor stratigraphy provides a robust and reliable method for delineating petit-spot volcanism and assessing its geological impact on the abyssal seafloor.

## Introduction

Petit-spot volcanism occurs when carbon dioxide-rich magma beneath a plate erupts along fractures created by flexuring of the plate before subduction^1,2^. Because of the specificity of the eruption process and erupted magma, petit-spot volcanism is not only a potential source of carbon dioxide emissions to the oceanosphere and atmosphere^3^ but also has the potential to modify the oceanic plate from the lower-most part to the topmost sediments^2,4–7^. To quantitatively identify the extent of the impact of these events on the Earth’s material cycle, we must know how spatially widespread the activity of petit-spot magma is.

Conventional methods for identifying the distribution of petit-spot volcanoes involve the use of a vessel-equipped multibeam acoustic echo sounder (MBES) to observe the topography and backscatter intensity of the seafloor^1,8^. For example, petit-spot volcanic activity has been reported in the western North Pacific Ocean in four areas^2^. In each of these areas, the size of individual volcanic edifices is an order of magnitude smaller than those formed by other igneous activities such as hotspot volcanism^8^. However, multiple volcanic edifices are distributed only within a certain area and form clusters, forming so-called “petit-spot volcanic fields”. Furthermore, it has been reported that petit-spot lava is injected horizontally into sedimentary layers^9^ and that lava outcrops exist even on flattened seafloors^10^where no topographic highs have formed. These observations indicate that petit-spot volcanic fields extend beyond the area recognized only via the topography.

Subbottom profiling (SBP) is a standard instrument installed on modern research vessels and is widely used in deepwater settings to examine the characteristics of sub-seafloor geological structures^11,12^owing to its simplicity and ease of operation. This method provides dense and continuous profiles of sub-seafloor structures, such as sedimentary layers, typically achieving penetration depths of up to 100 m. Therefore, compared to multichannel seismic (MCS) reflection surveys^13^, SBP offers stratigraphic analysis with higher spatial resolution in shallower regions. This is attributed to its use of higher acoustic frequencies (ranging from 2 to 21 kHz), which makes SBP a complementary tool to MCS in sub-seafloor imaging. Another advantage of SBP over MCS is its ability to be operated simultaneously with MBES. This allows for the construction of integrated datasets in which seafloor surface topography and backscatter intensity can be directly correlated with sub-seafloor structures, enabling more comprehensive geological interpretations.

**Fig. 1:**
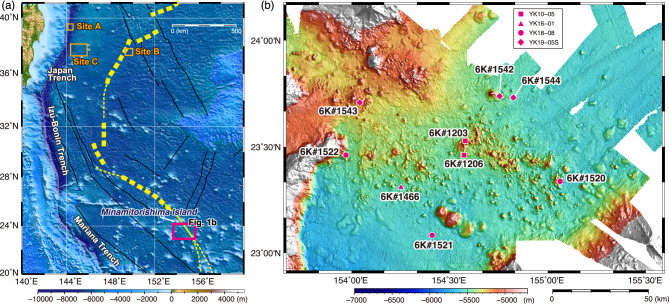
Map of the western North Pacific showing the locations of petit-spot volcanic fields (**a**) and detailed bathymetry around the Minamitorishima petit-spot field (**b**). The dotted yellow line shows the eastern edge of the outer rise of the western Pacific trench–subduction systems from Hirano et al.^20^. The black lines are the fracture zones defined by Nakanishi and Winterer^17^. All the maps were created using the Generic Mapping Tools (GMT, ver. 5.4.5; https://www.generic-mapping-tools.org). The global bathymetric data for (**a**) from ETOPO1 (https://www.ngdc.noaa.gov/mgg/global/). High-resolution bathymetric data obtained via vessel-equipped MBES for (**b**) are from this study (see Materials and Methods).

By applying SBP to investigate the stratigraphic features of the seafloor around petit-spot volcanic fields, we can identify not only the geological structure of the region related to lava eruption but also the extent of lava injection into near surface sedimentary layers that are not visible as topographic features. Moreover, acoustic surveys using deep-sea vehicles, such as an autonomous underwater vehicle (AUV) or a remotely operated vehicle (ROV), are widely used to reveal seafloor topography and sub-seafloor stratigraphy with exceptionally high spatial resolutions^14,15^. This approach also contributes to the visualization of petit-spot volcanic edifices, particularly in cases where vessel-based observations need to be ground truthed. In this report, we compile multi-scale SBP data obtained from both vessel-based and submersible-based observations around Minamitorishima (Marcus) Island in the western North Pacific (Fig. 1). Additionally, we verified these data through in-situ observations, using a human occupied vehicle (HOV) named *SHINKAI 6500*. Based on these investigations, we propose a protocol for determining regions of petit-spot volcanic fields.

## Materials and methods

### Geological background

The petit-spot volcanic field, situated approximately 100 km southeast of Minamitorishima Island in the western Pacific (Fig. 1), lies within Cretaceous seamounts, forming a segment of the Marcus–Wake Seamount chains within the broader western Pacific Seamount Province^16^. This area represents the oldest expanse of the Pacific plate, with seafloor spreading dating back 160 million years^17^. Post-spreading volcanism of alkaline magma was also recognized^18,19^. However, the age of all these volcanisms related to the formation of large seamounts and the underlying seafloor is significantly older than that of petit-spot volcanism. Investigations into the petrography, geochemistry, and geochronology of petit-spot basalts and zircons collected during the cruise YK10-05 of *R/V Yokosuka*^20^ suggest regional magma ascension from the asthenosphere along the flexed plate. This process is estimated to have occurred within the past 3 million years, correlating with the region’s proximity to the Mariana subduction system and the presence of the outer-rise bulge^20^. These findings are indicative of a dynamic geological context and complex interplay of forces, as evidenced by the ascent of magmas, underscoring the complexity of volcanic activity within the petit-spot volcanic field. Moreover, the southern portion of this region borders the dense ferromanganese nodule field reported by Refs^21,22^. Therefore, further detailed geological investigations will not only elucidate the geological processes shaping the region but also provide valuable insights into the broader tectonic dynamics and their effects on seafloor geology and the deep-ocean environment in the western Pacific.

## Field observations

**Fig. 2:**
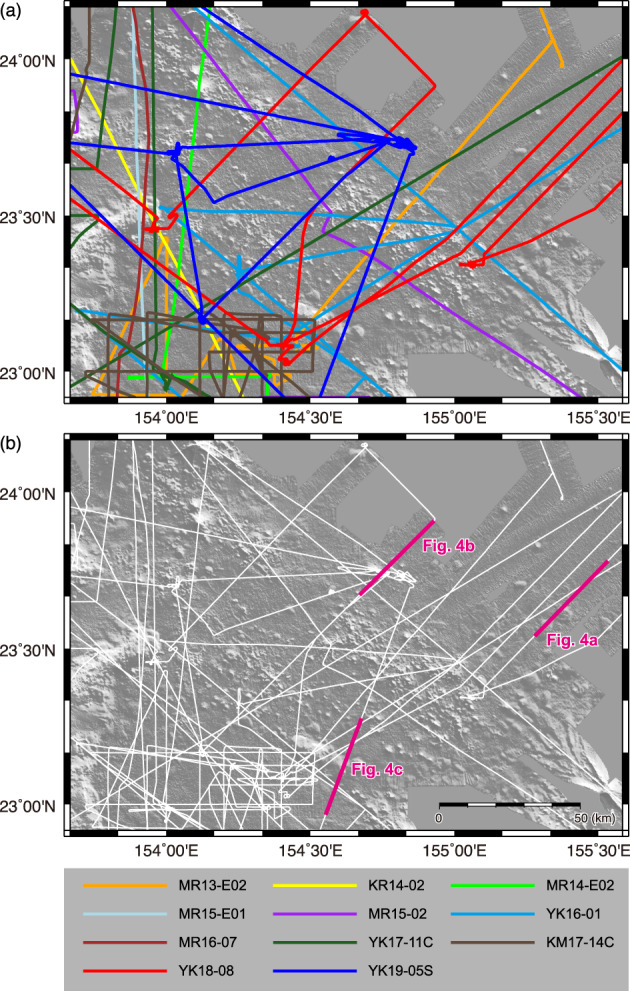
Map illustrating (**a**) vessel-equipped subbottom profiling (SBP) tracks of eleven research cruises and (**b**) the locations of the representative SBP profiles shown in Fig. 4. The bathymetric map shown in the background is the same as that in Fig. 1b. All the maps were created using the Generic Mapping Tools (GMT, ver. 5.4.5; https://www.generic-mapping-tools.org).

To investigate the detailed geological structure around the petit-spot volcanic field approximately 100 km southeast of Minamitorishima Island (hereafter termed the “Minamitorishima petit-spot field”) to determine the distribution of the area influenced by petit-spot magma injection, we focus on acoustic data obtained by vessel-equipped MBES and SBP during eleven research cruises in this region (Fig. 2; Table 1). The western part of the research area was covered by five main research cruises (KR14-02, MR14-02, MR15-E01, MR16-07, and YK17-11C) to constrain the distribution of basement rocks constituting of large seamounts, the Marcus–Wake Seamount chains, Minamitorishima Island and the Takuyo Daigo Seamount. Geological transitions from the Minamitorishima petit-spot field to the dense ferromanganese nodule field reported by Refs.^21,22^. were investigated on the basis of data obtained during cruises MR13-E02 Leg 2, MR14-E02, and KM17-14C. The boundaries between the southeastern part of the Minamitorishima petit-spot field and large seamounts of the Marcus–Wake Seamount chains or the northeastern portion of the Minamitorishima petit-spot field and the sedimentary seafloor were constrained by data from cruises MR13-E02 Leg 2, MR15-02, YK16-01, and YK17-11C. MBES and SBP data obtained during cruises YK18-08 and YK19-05S also contribute to defining seafloor facies across the lava field. The results of the analysis of the MR13-E02, KR14-02, MR14-E02, MR15-E01, and MR15-02 data in this region reported by Ref^23^. were re-examined in this study.

**Table 1:** List of reserch cruises conducted in this study.

Vessel name	Cruise No.	Observations					
		Vessel-based Acoustic Surveys			Submersible Dives		
		MBES	SBP	Ref.	Rock Sampling	MBES	SBP
R/V Mirai	MR13-E02	◯	◯	23	–	–	–
	MR14-E02	◯	◯	23	–	–	–
	MR15-E01	◯	◯	23	–	–	–
	MR15-02	◯	◯	23	–	–	–
	MR16-07	◯	◯	*	–	–	–
R/V Kairei	KR14-02	◯	◯	23	–	–	–
R/V Kaimei	KM17-14C	◯	◯	*	–	–	–
R/V Yokosuka	YK10-05	◯	–	*	◯	–	–
	YK16-01	◯	◯	*	◯	◯	◯
	YK17-11C	◯	◯	*	–	–	–
	YK18-08	◯	◯	*	◯	◯	◯
	YK19-05S	◯	◯	*	◯	◯	◯

*SBP data were examined in this study.

**Fig. 3:**
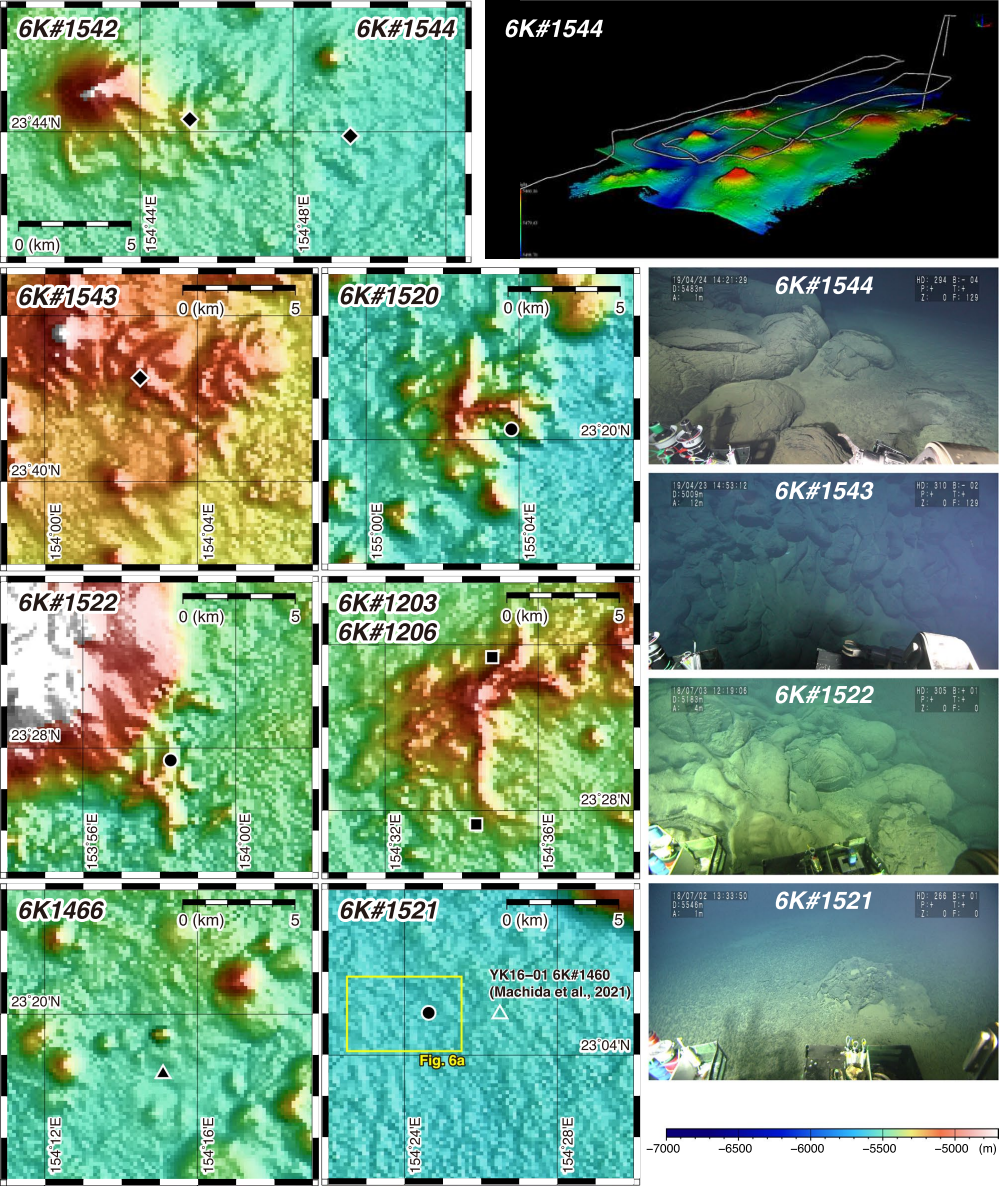
Detailed bathymetric map of petit-spot volcanoes, photographs of outcrops, and three-dimensional whale view map. Photographs were taken by the dives of *SHINKAI 6500*. All the two-dimensional maps were created using the Generic Mapping Tools (GMT, ver. 5.4.5; https://www.generic-mapping-tools.org). Bathymetric data obtained via vessel-equipped MBES for the two-dimensional maps are from this study (see Materials and Methods). Bathymetric data for the three-dimensional map of dive 6K#1544 (upper-most right panel; Kaneko et al.^24^) obtained along survey track line (white line) by deep-sea MBES equipped with *SHINKAI 6500*.

To observe lava outcrops and collect samples of petit-spot volcanoes, nine dives of the *SHINKAI 6500* submersible were conducted during cruises YK10-05, YK16-01, YK18-08, and YK19-05S (Fig. 3; Table 1). Detailed lithological descriptions, chemical compositions, and ages of the samples were reported previously^7,20^. The deep-sea SBP and deep-sea MBES were equipped with a *SHINKAI 6500* for all dives, except for those of cruise YK10-05. The results of deep-sea MBES surveys were reported by Ref^24^.

## Acoustic survey via Subbottom profilers

Profiles of the subseafloor structure of southeast of Minamitorishima Island were recorded by three different vessel-equipped subbottom profilers (Table 2): 3300-HM (EdgeTech Inc.), Bathy2010 (SyQwest Inc.) and TOPAS PS18 (Kongsberg Inc.). The 3300-HM, which was used during cruises YK16-01, YK17-11C, YK18-08 and YK19-05S, has a frequency of 2–16 kHz and a chirp waveform. Bathy2010, which was used during cruises MR13-E02 Leg2, MR14-E02, MR15-02, MR15-E01, MR16-07 and KR14-02, has a centre frequency of 3.5 kHz. TOPAS PS18, which was used only during cruise KM17-14C, has a frequency range of 15–21 kHz (primary) and uses a parametric method. These specifications of instruments are useful for shallow geological exploration in the research area. In addition, the *SHINKAI 6500* is equipped with a deep-sea subbottom profiler, StrataBox (Syquest, Inc.), which was used in all dives during cruises YK18-08 and YK19-05S (Table 2). The specifications were a strata resolution of 6 cm with 40 m of bottom penetration and a centre frequency of 10 kHz.

**Table 2:** Configurations of subbottom profiling.

Vessel name	Vessel-equipped		Submersible-equipped	
	Model	Frequency (kHz)	Model	Frequency (kHz)
R/V Mirai	Bathy2010	3.5	–	–
R/V Kairei	Bathy2010	3.5	–	–
R/V Kaimei	TOPAS PS18	15–21 (Primary)	–	–
R/V Yokosuka	3300-HM	2–16	Strata Box	10

The spatial resolution of vessel-equipped and deep-sea SBP was evaluated in terms of two components: vertical resolution (instrumental resolution) and horizontal resolution (determined by navigation speed). Assuming a representative water depth of 6,000 m, sound velocity in water of 1,500 m/s, speed of 10 knot for the surface vessel or 1 knot for the deep-sea vehicle (*SHINKAI 6500*), and a shot interval of 2 Hz, the estimated vertical resolutions were approximately 8 cm and 6 cm for the vessel-equipped and deep-sea SBP, respectively. The estimated horizontal resolutions along the track line were approximately 40 m and 1 m for the vessel-equipped and deep-sea SBP, respectively.

## Definitions of acoustic facies identified by vessel-equipped Subbottom profilers

**Fig. 4:**
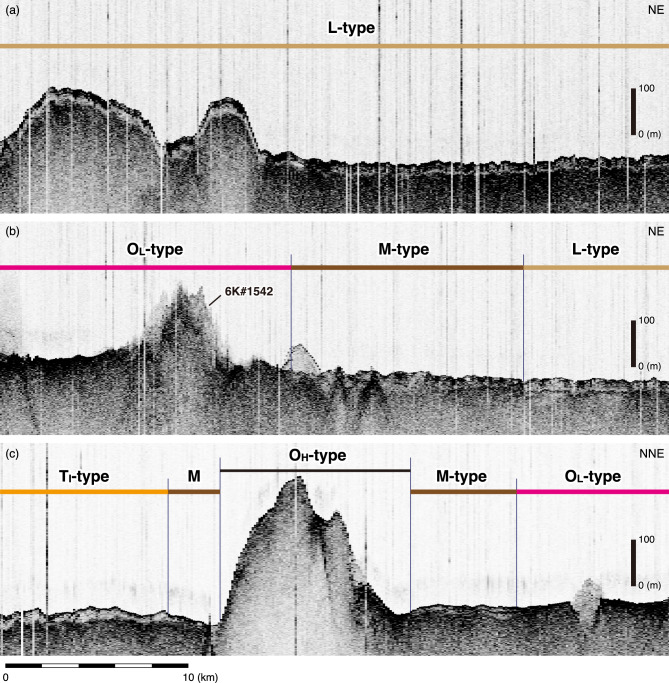
Representatives of the results of vessel-equipped subbottom profiling, illustrating features of the facies types obtained in this study. The L-type facies overlies the T-type facies.

**Fig. 5:**
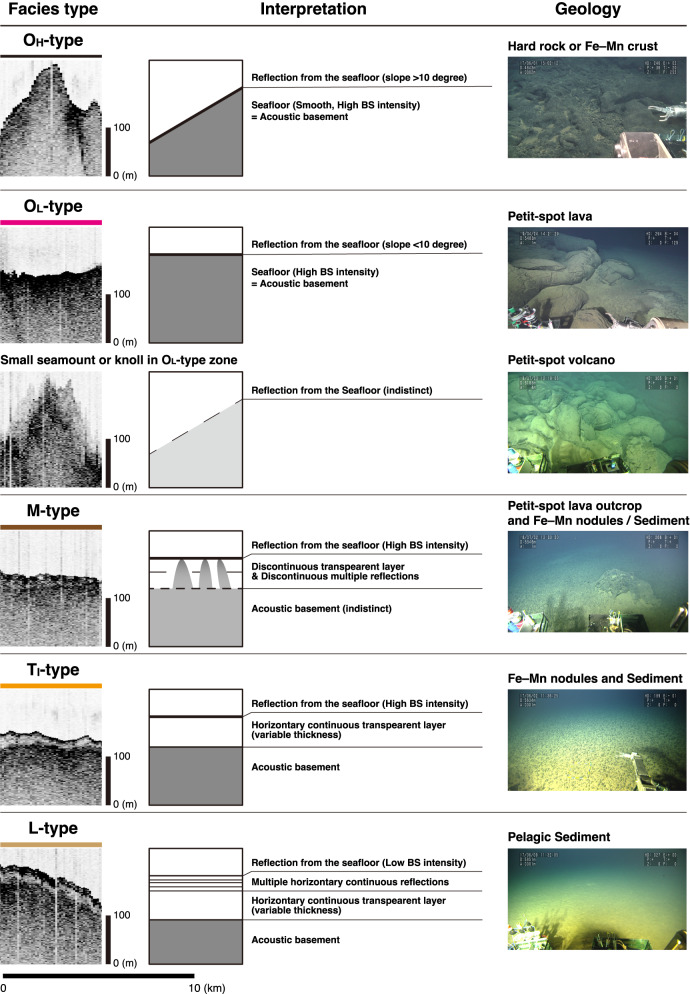
Summary for echo facies types determined using vessel-equipped subbottom profiling. Representative cross-sections for each facies type are shown in Fig. 4. Seafloor photographs for the O_L_- and M-types are the same as those shown in Fig. 3, while those for the O_H_-, T_I_-, and L-types are from Ref^22^.

All SBP data were visualized and processed using the SonarWiz8 software (https://chesapeaketech.com). We classified the cross-section determined by the vessel-equipped SBP into three acoustic facies by following the criteria defined by Ref^23^. O-type facies are acoustically opaque (Figs. 4 and 5). T-type facies are acoustically transparent, with no horizontally continuing reflection between the upper-most reflection from the seafloor and the underlying acoustic basement (Fig. 5). Irregular (T_I_) type facies^23^ have an undulating surface (seafloor), generally parallel to the topography of the acoustic basement (Figs. 4 and 5). L-type facies are characterized by several horizontal continuous layers immediately below the seafloor. When L-type facies are observed, the T-type facies is always sandwiched between the L-type facies and the acoustic basement (Figs. 4 and 5), as described by Ref^23^.

In particular, in the study area, the O-type facies was further subdivided into two different cases depending on the slope gradients of the seafloor (Figs. 4 and 5). The O_L_-type facies corresponds to regions with a low slope gradient, whereas the O_H_-type facies corresponds to regions with a high slope gradient. We distinguish both facies by a boundary gradient with a slope of 10 degrees, because slopes exceeding this threshold include high-backscatter regions covered by ferromanganese crusts^22^.

Moreover, we identified a zone of facies exhibiting discontinuous reflections in the transparent layer. This zone also includes small discontinuous regions exhibiting O-type facies-like features. These features occur at spatial intervals ranging from several hundred of metres to several kilometres. Based on these characteristics, where multiple facies coexist, as schematically shown in Fig. 5, we define this zone as a multiple-type (M-type) facies. The slope gradient for regions with M-type facies is less than 10 degrees, as is the slope gradient for L- and T_I_-type facies.

In summary, we use the following strategy to distinguish stratigraphic facies. If we observe multiple continuous reflections in the upper part of the transparent layer, those regions are classified as L-type facies. In contrast, regions exhibiting no reflections are classified as T_I_-type. As previously mentioned, O_H_- and O_L_-type facies are found in regions where the acoustic basement is exposed on the seafloor, and they are differentiated on the basis of a slope of 10 degrees. Regions where multiple facies are observed at short spatial intervals are classified as M-type facies.

## Results

**Fig. 6:**
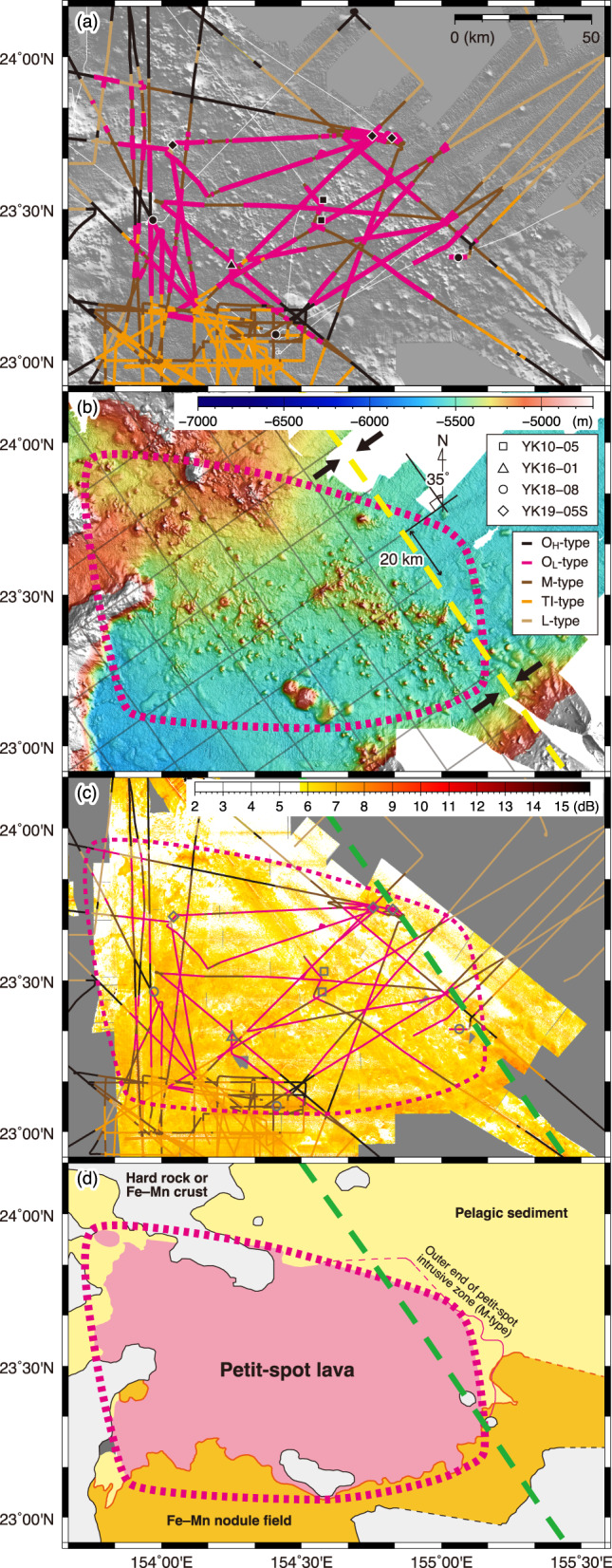
Distribution of acoustic facies and petit-spot volcanic fields. The colour for each acoustic facies along the subbottom profiling tracks in (**a**) and (**c**) is the same as that in Figs. 4 and 5. A tentative interpretative map of the study on the basis of the facies distribution, topography, and acoustic backscatter intensity is presented in (**d**). White lines in (**a**) represent tracks without profiling. Dotted pink lines in (**b**), (**c**), and (**d**) represents the identified region of the petit-spot volcanic field on the basis of the distribution of the O_L_-type facies. Dotted yellow and green lines in (**b**), (**c**), and (**d**) show the eastern edge of the outer rise of the western Pacific trench–subduction systems identified by Hirano et al.^20^. Black arrows represent the orientation of compressional stress, which is perpendicular to the axis of least compressive principal stress (σ_3_) due to the concave flexure of lithosphere. Gray grid lines in (b) are oriented along and perpendicular to the eastern edge of the outer rise (the axis of σ_3_). All the maps were created using the Generic Mapping Tools (GMT, ver. 5.4.5; https://www.generic-mapping-tools.org). The global bathymetric data for (**a**) from ETOPO1 (https://www.ngdc.noaa.gov/mgg/global/). High-resolution bathymetric data obtained via vessel-equipped MBES for (**b**) are from this study (see Materials and Methods). The acoustic backscatter intensity data shown in (**c**) are from Machida et al.^22^.

In the study area, what is particularly notable is the large-scale distribution of O_L_-type facies in the central region (Fig. 6). O_H_-type facies is especially prominent along survey lines crossing seamounts. We observed only T_I_-type facies in the southern part of the study area. In contrast, L-type facies is extensively distributed in the northeastern part of the study area and also occurs between seamounts in the northwestern part. M-type facies typically appears at the boundary region between different facies (e.g., O- and L-types or O- and T_l_-types), as well as in the interregion of each facies (Figs. 4 and 6).

**Fig. 7:**
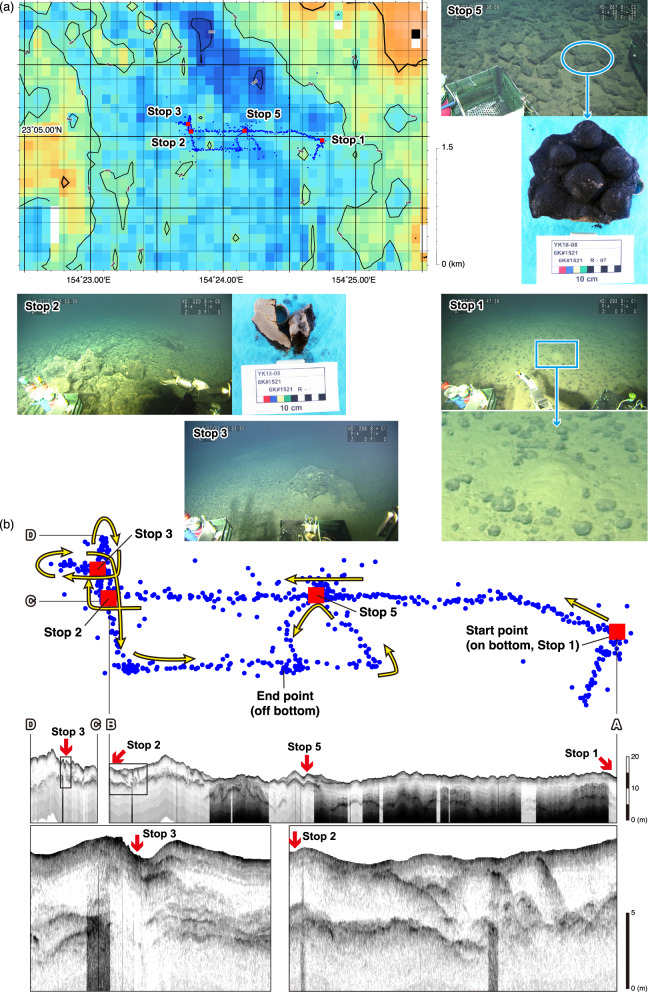
Summary of the megascopic and acoustic surveys during dive 6K#1521. (**a**) Blue dots indicate the position of the submersible *SHINKAI 6500* every 10 s. Red squares mark the stop points for detailed seafloor observations and rock sampling. A small outcrop of petit-spot lava was recognized at Stop 3 (Mikuni et al.^7^). Photographs of the sample show sedimentary rock collected from Stop 2 and overgrown ferromanganese nodules collected from Stop 5. (**b**) The profiles of sub-seafloor structure along the dive track, obtained via deep-sea SBP, are shown. The position of the submersible *SHINKAI 6500* (blue dots), same as in (**a**), is also indicated. Yellow arrows show the movement directions of the submersible during the dive. The slight misalignment of the position of Stop 5 between the dive track and SBP profile is due to subtle variations in the submersible’s speed or slight meandering. The black boxes in the middle two SBP profiles correspond to the bottom two close-up SBP profiles. The bathymetric map in (**a**) was created using the Generic Mapping Tools (GMT, ver. 5.4.5; https://www.generic-mapping-tools.org). High-resolution bathymetric data obtained via vessel-equipped MBES are from this study (see Materials and Methods).

To investigate the stratigraphic features of M-type facies, we conducted acoustic surveying via a deep-sea subbottom profiler equipped with on the *SHINKAI 6500* during dives 6K#1521, 6K#1542, and 6K#1544. Figure 7 shows the surface geology and subsurface stratigraphy along the track line of dive 6K#1521 as the representative of M-type facies, i.e., the case for the transition from the ferromanganese nodule field to the petit-spot volcanic field. Using the deep-sea subbottom profiler equipped on the *SHINKAI 6500*, observations successfully revealed the bedrock and dikes of petit-spot lava intruding into the underlying sediment beneath the seafloor. We previously reported that a small outcrop of petit-spot lava was recognized in the ferromanganese nodule field at Stop 3 (Ref^7^.; Fig. 7a). Around Stop 3, the “deepest” reflector (acoustic basement) abruptly becomes shallower and extends up to the seafloor (Fig. 7b). In contrast, at Stop 2, where sedimentary rock outcrops were identified (Fig. 7a), the “shallow” reflectors above the deepest reflector rise close to the seafloor (Fig. 7b). At the arrival point (Stop 1), the deepest reflector is located at a depth of approximately 10 m, but it becomes shallower near Stop 5. West of Stop 5, most of the deepest reflector lies at a depth of 5 m or less.

## Discussion

Our results from the survey of vessel-equipped SBP show that the O_L_-type facies are widely distributed in the central region of the area (Fig. 6a). During submersible dive 6K#1544, typical O_L_-type facies were observed in a deep-sea plain, and many lava flows were confirmed through both deep-sea MBES and megascopic observations (Ref.^24^; Fig. 3). Similarly, during submersible dive 6K#1466, lava outcrops were also confirmed in flat areas^7^. Moreover, other small seamounts within the widespread O_L_-type facies are considered petit-spot volcanoes on the basis of our submersible dives (Ref.^7^; Figs. 3 and 6). Therefore, the O_L_-type facies is a critical acoustic feature indicating the distribution of petit-spot lava outcrops on the seafloor. We also recognize that acoustic reflections from the surface of petit-spot volcanoes are scattered (Figs. 4b and 5). We consider that the high roughness of the surface of petit-spot volcanoes due to the distribution of fresh lava flows, as shown by our submersible dives (Fig. 3), is the cause of acoustic scattering. In contrast, the O_H_-type facies, which are primarily found along the slopes of large seamounts located in the eastern (Takuyo-Daigo Seamount) and northeastern (Minamitorishima) parts of the area (Figs. 5 and 6a), have smooth surfaces due to the coverage of ferromanganese crust (Figs. 4c and 5; Ref.^22^) that contrasts with the surfaces of petit-spot volcanoes. We consider that the O_H_-type facies with smooth topography can be an indicator of non-petit-spot seamounts.

The T_I_-type facies is observed in the southern part of the area, whereas the L-type facies is the major facies of the northern (particularly northeastern) part of the area. Considering the distribution of L-type facies along with the results of piston core sampling reported by Ref.^23^, it is suggested that non-rare earth elements plus yttrium (REY)-rich hemipelagic sediments are present in the northeastern part of the study area. In contrast, the region containing the T_I_-type facies has a reflection intensity greater than 5.72 dB of backscatter data reported by Ref.^22^ (Fig. 6c), which corresponds to areas where a dense distribution of ferromanganese nodules is found. Between the L- or T_I_-type facies and the O_L_-type facies, there is an M-type facies. Investigations by the *SHINKAI 6500* submersible dives via deep-sea SBP at each representative site of the M-type facies next to the L-type (dive 6K#1544) or T_I_-type (dive 6K#1521) facies confirmed that petit-spot lava intruded into either non-REY-rich hemipelagic sediments or ferromanganese nodule fields (Figs. 3 and 7).

In the vicinity of large seamounts, low-viscosity alkaline basaltic lava—capable of flowing over long distances (up to ~ 50 km; Refs.^25–27^)—may form reflective interfaces within sedimentary layers. However, aside from petit-spot volcanism, all igneous activity in the broader Western Pacific Seamount Province is older than 100 Ma^16,28,29^. At ODP Site 801^18^, sedimentary layers exceeding 400 m in thickness are superimposed on these ancient igneous materials (e.g., lava flows). Due to the shallow acoustic penetration limit of SBP, such deep-seated structures should not be detectable. An exception is the overprinting caused by the alkaline basalt eruption that occurred between 40 and 58 Ma on the northwestern slope of Minamitorishima Island^30^. However, we observed that the L-type facies along the southern slope of Minamitorishima Island borders the O_L_-type facies (near 24°N and 153°30’E, as shown in Fig. 6a). This finding suggests the presence of young lava intrusions within the sedimentary layers, rather than shallow reflections of lava flow originating from Minamitorishima Island. Fundamentally, the shallow acoustic reflections obtained in the M-type facies correspond to young intrusive petit-spot lava, as traced via deep-sea SBP during dives 6K#1521, 6K#1542, and 6K#1544. Therefore, the M-type facies, located between O_L_-, L-, and T_I_-type facies, indicates the outer boundary zone of the petit-spot volcanic field and can be defined as a transitional area where volcanic activity gradually appears or disappears.

Based on the results of our investigation, we provide a tentative interpretative map of the study area (Fig. 6d). Facies boundaries outside the SBP survey lines were determined by tracing the lateral extension of boundaries observed on the SBP survey lines, based on changes in features of both backscatter intensity (e.g., intensity contrast) and topography (e.g., lineaments and/or inflection of slope gradients). This map further clarifies the distribution of the contrasting geological regions in the study area, as previously described. The pelagic sediment region (L-type facies) dominates in the northern part, while the ferromanganese nodule field (T_I_-type facies) dominates in the southern part. We consider that this basic framework of geological distribution relates to oceanographic settings in terms of deep-sea current flowing into the study area from the south, originating from the Lower Circumpolar Deep Water, as discussed by Ref ^31^.

The interpretative map also illustrates the relationship between two primary regions—pelagic sediment and ferromanganese nodules—and the position of the petit-spot volcanic fields. Building on this, we propose criteria for determining petit-spot volcanic fields. The most critical criterion indicating the distribution of petit-spot lavas is the concurrent observation of high backscatter intensity obtained from MBES data and the O_L_-type facies obtained from SBP data. Definitive evidence for petit-spot volcanism can be provided by ground truthing observations of a small seamount or knoll with an acoustically scattered slope surface situated in the region indicating high backscatter intensity and the O_L_-type facies. The distribution of the O_L_-type facies constrains the exact dimensions of the petit-spot volcanic field. In light of our results, we suggest that the distribution of the M-type facies should be taken into account when assessing the extent of the overall impact of petit-spot volcanism on seafloor geology. As shown in Fig. 7, at the seafloor where the petit-spot lava intrusion took place (recognized as M-type facies) in the southern boundary zone with the ferromanganese nodule field, we observed mounds resembling mud volcanoes (Stop 1), intrusion of sedimentary rock (Stop 2) continuing to the “shallow” reflectors, and plate-like aggregates (Stop 5) because several ferromanganese nodules had overgrown (referred to as “encrustation”). Although detailed consideration of the causal link between these geological features observed on the seafloor and petit-spot lava intrusion is needed in future studies, the M-type facies in this zone, which confirmed high backscatter intensity (Fig. 6c) and geological impact (Fig. 7a), can be included in the petit-spot volcanic field. In contrast, in the northeastern connection zone with the pelagic sediment (Fig. 6d), the presence of M-type facies suggests that petit-spot lava intrusion within the sediment layer is likely to have occurred. However, we did not observe other criteria such as high backscatter intensity nor confirm ground-truth observations for geological impact. Therefore, the M-type facies in this region is not included within the petit-spot volcanic field and is instead represented by a boundary line (Fig. 6d). It is important to consider the uncertainties associated with the geological information suggested by the acoustic data when assessing the extent of the petit-spot volcanic fields.

Direct observations, whether through cameras or the human eye, remains the most reliable method for confirming and documenting volcanic formations. However, their abilities to continuously trace lava outcrops across the full extent of seamount or knoll slopes—or across topographically flat regions beyond these features—are inherently limited. In contrast, although vessel-equipped SBP is constrained to imaging shallow sedimentary layers, this technique offers a comprehensive and efficient means of mapping widespread lava eruptions and intrusions. One of the key advantages of vessel-equipped SBP is its ability to detect lava bodies that have not reached or fully exposed themselves at the seafloor surface. In such cases, outcrops may be undetectable by direct observation using submersibles. However, deep-sea SBP can identify subsurface reflections potentially indicative of lava intrusions within sediment layers. By carefully examining locations where these shallow reflections approach the seafloor, we can more effectively locate lava outcrops—as demonstrated by dive 6K#1521 in this study (Fig. 7). Therefore, direct observation of the seafloor provides definitive evidence for the interpretation of acoustic data. Essentially, acoustic observations and direct observations are complementary.

Considering the complex interactions inherent in plate metamorphism, such as reactions between petit-spot melt and wall rock and the movement of volatile substances within the plate^2^ it is conceivable that some melt could potentially freeze within the plate, without ever reaching the seafloor surface. Therefore, it is important to recognize that the distribution of magma exposed on the seafloor surface provides a lower limit to the magnitude of the impact of petit-spot melt on the entire plate. The integrated SBP approach is particularly powerful because it allows for comprehensive detection including small-scale features that might not be fully captured by conventional surveys, which involve greater resource demands and more detailed operational requirements, such as MCS. A wide-area exhaustive survey using the protocol proposed in this study can effectively guide targeted MCS investigations, particularly for imaging deeper structures, thereby achieving broader survey coverage while making more efficient use of available research resources. This study demonstrates that the integrated use of seafloor backscatter intensity and subseafloor stratigraphy, obtained from both vessel-equipped multibeam echo sounders and subbottom profilers, provides a cost- and time-effective strategy with both wide coverage and high spatial resolution for delineating the surface distribution of petit-spot lava.

In summary, the O_L_-type facies is a key indicator of petit-spot lava, with acoustic reflections from these facies revealing the spatial extent of lava eruptions. Additionally, we identified the M-type facies as an important feature indicating the transition between different facies and the boundary of the petit-spot volcanic field. The results of our SBP surveys, complemented by submersible dive observations, show that SBP is particularly effective for detecting subsurface lava intrusions that are not easily visible through direct observation. The integration of vessel-based and submersible-based observations enables the detection of smaller-scale volcanic features and offers a more comprehensive understanding of volcanic processes. The proposed strategy—using SBP for broad seafloor coverage, followed by targeted MCS for deeper imaging—offers a cost- and time-effective way to map both shallow and deep structures. Our protocol lays the groundwork for identifying the extent and impact of petit-spot volcanism within the entire subducting plate.

## Data Availability

The SBP and MBES data used in the study are available at Data and Sample Research System for Whole Cruise Information (DARWIN) in Japan Agency for Marine-Earth Science and Technology (JAMSTEC) via the following list of DOI for each cruise. MR13-E02 Leg2: https://doi.org/10.17596/0001859; KR14-02: https://doi.org/10.17596/0001205; MR14-E02: https://doi.org/10.17596/0002391; MR15-02: https://doi.org/10.17596/0002392; MR15-E01 Leg2: https://doi.org/10.17596/0002394; MR15-E01 Leg3: https://doi.org/10.17596/0002395; YK16-01: https://doi.org/10.17596/0002416; MR16-07: https://doi.org/10.17596/0002398; YK17-11C: https://doi.org/10.17596/0002424; KM17-14C: https://doi.org/10.17596/0002366; YK18-08: https://doi.org/10.17596/0001706; YK19-05S: https://doi.org/10.17596/0002034.
